# Ten-Year Anniversary of the *International Journal of Neonatal Screening*: Revisiting Its Scope

**DOI:** 10.3390/ijns11020042

**Published:** 2025-06-04

**Authors:** Ralph Fingerhut, Peter C. J. I. Schielen

**Affiliations:** 1 SYNLAB MVZ Weiden, Zur Kesselschmiede 4, 92637 Weiden, Germany; ralph.fingerhut@synlab.com; 2 International Society for Neonatal Screening, Reigerskamp 273, 3607 HP Maarssen, The Netherlands

The 10th anniversary of the *International Journal of Neonatal Screening* (*IJNS*) was celebrated on 25 March 2025, during the 13th Regional European Meeting of the International Society of Neonatal Screening (ISNS) in Luxembourg.

*IJNS* has established itself as a leading platform for research on all aspects of neonatal screening and is indexed in Scopus, ESCI (Web of Science), PubMed, PMC, Embase, and other databases. It has published over 500 papers from more than 2000 authors and has been reviewed by over 400 reviewers. In recognition of its rapid growth, *IJNS* received its first Impact Factor (IF) in 2023, which currently stands at 4.0 (CiteScore of 6.7).

Since its founding 10 years ago, we have gained a deeper understanding of the typical submissions to the journal. Based on this knowledge, and considering the rapidly evolving world of neonatal screening, the Editors-in-Chief, Associate Editors, and Editorial Board of *IJNS* have decided that this milestone provides a valuable opportunity to re-evaluate the journal’s scope, which was initially somewhat limited and vague.

A major clarification was made regarding the acceptance of manuscripts, extending beyond laboratory tests to encompass all core elements of neonatal screening, from parental invitations to diagnosis and treatment. The subjects now covered are described in greater detail.

At times, the Editorial Board has struggled to determine whether a manuscript falls within the scope, such as those on subjects like hearing loss screening using otoacoustic emission (OAE) or Critical Congenital Heart Disease (CCHD) screening through pulse oximetry. This is now made more explicit.

*IJNS* also wishes to clarify that papers in the field of prenatal screening are acceptable when they are related to current neonatal screening (e.g., prenatal screening for medium-chain acyl-coenzyme A dehydrogenase deficiency (MCADD), which is an established neonatal screening disorder).

The year 2024 was a significant year for neonatal screening, as, during the World Health Organization Assembly (WHA77), a resolution was adopted for the first time that specifically mentioned neonatal screening. The wording of that resolution is as follows:

*“() to consider implementing a universal newborn screening program, including comprehensive birth defect screening, looking at specific needs and considerations for the diagnosis, management and long-term care of children with birth defects.”*

*(https://apps.who.int/gb/ebwha/pdf_files/WHA77/A77_ACONF5-en.pdf, p. 5, accessed on 28 April 2025)*

The WHO recognizes a wider range of birth defects than those commonly covered by the screening panels of neonatal screening programs. These include disorders such as CCHD (as previously mentioned), club feet (talipes), and hip dysplasia, among others. To align with the disorders highlighted in this significant resolution from arguably the most influential health organization in the world, we have clarified this further in the renewed scope. However, it remains essential that submitted manuscripts maintain a clear screening perspective.

Also, we thought it would be wise to share the mission and vision of the ISNS, which, while not as clearly defined in 2015, is now, rightfully, one of the key principles shaping the scope of *IJNS*.

Finally, in just ten years, *IJNS* has built quite a strong reputation. Several national professional organizations in the field of neonatal screening have expressed their support and are pleased to collaborate with *IJNS*, promoting it as the journal of choice to disseminate the work of these national organizations. Notably, the American Association of Public Health Laboratories, German Society for Neonatal Screening, French Society for Neonatal Screening, Japanese Society for Neonatal Screening, and the UK Newborn Screening Laboratory Network are currently affiliated with *IJNS*.

Renewing and refining the scope of our journal is not a decision we make lightly. This updated scope was developed by the Editors-in-Chief and Associate Editors of *IJNS*, approved by the council of the ISNS (the owner of *IJNS*), and ultimately endorsed by the Editorial Board of *IJNS*.

With this, we are confident that *IJNS* is ready for the next ten years, with the ambition to remain the world’s leading journal for neonatal screening. For this, we rely on the continued support of you, the readers, and future contributors to *IJNS*.

On behalf of the Editors-in-Chief, Associate Editors, and editors of *IJNS*, we extend our heartfelt thanks for your support.

